# Cardiac and Vascular Synergic Protective Effect of *Olea europea* L. Leaves and *Hibiscus sabdariffa* L. Flower Extracts

**DOI:** 10.1155/2015/318125

**Published:** 2015-06-09

**Authors:** Matteo Micucci, Marco Malaguti, Tullia Gallina Toschi, Giuseppe Di Lecce, Rita Aldini, Andrea Angeletti, Alberto Chiarini, Roberta Budriesi, Silvana Hrelia

**Affiliations:** ^1^Department of Pharmacy and Biotechnology, Alma Mater Studiorum, University of Bologna, Via Belmeloro 6, 40126 Bologna, Italy; ^2^Department for Life Quality Studies, Alma Mater Studiorum, University of Bologna, Corso d'Augusto 237, 47900 Rimini, Italy; ^3^Department of Food Science, University of Bologna, Via Fanin 44, 40100 Bologna, Italy; ^4^Department of Specialistic, Diagnostic and Experimental Medicine, Section of Nephrology, University of Bologna, S. Orsola Hospital, Via Massarenti 9, 40138 Bologna, Italy

## Abstract

This study was aimed at investigating the cardiovascular effects of an *Olea europea* L. leaf extract (OEE), of a *Hibiscus sabdariffa* L. flower extract (HSE), and of their 13 : 2 w/w mixture in order to assess their cardiac and vascular activity. Both extracts were fully characterized in their bioactive compounds by HPLC-MS/MS analysis. The study was performed using primary vascular endothelial cells (HUVECs) to investigate the antioxidant and cytoprotective effect of the extracts and their mixture and isolated guinea-pig left and right atria and aorta to evaluate the inotropic and chronotropic activities and vasorelaxant properties. In cultured HUVECs, OEE and HSE reduced intracellular reactive oxygen species formation and improved cell viability, following oxidative stress in dose-dependent manner. OEE and HSE exerted negative inotropic and vasorelaxant effects without any chronotropic property. Interestingly, the mixture exerted higher cytoprotective effects and antioxidant activities. Moreover, the mixture exerted an inotropic effect similar to each single extract, while it revealed an intrinsic negative chronotropic activity different from the single extract; its relaxant activity was higher than that of each single extract. In conclusion OEE and HSE mixture has a good potential for pharmaceutical and nutraceutical application, thanks to the synergistic effects of the single phytochemicals.

## 1. Introduction

Hypertension is a chronical medical condition which represents a major risk factor for myocardial infarction, heart failure, stroke, peripheral arterial disease, and aortic aneurysm and is a cause of chronic kidney disease. This pathology often occurs along with metabolic syndrome and it is associated with a decreased life expectancy [1–3].

The central role in the pathogenesis belongs to the vascular endothelium. Vascular biology assumes a pivotal role in the initiation and perpetuation of hypertension and cardiovascular organ damage. Oxidative stress (ROS and RNS), inflammation, increased expression of redox-sensitive proinflammatory genes, cell adhesion molecules, and recruitment migration vascular dysfunction (T cells and B cells) are the primary pathophysiologic and functional mechanisms that induce vascular disease [4]. All these are closely interrelated and establish a deadly combination that leads to endothelial dysfunction (ED), vascular smooth muscle and cardiac dysfunction, hypertension, vascular disease, atherosclerosis, and cardiovascular diseases (CVD) [5]. Conventional pharmacological treatment for hypertension includes diuretics, angiotensin converting enzyme (ACE) inhibitors, angiotensin receptor blocker, *β*-receptors blockers, L-Type calcium channel blockers, and central *α*-receptors agonists.

International medical societies have long since introduced nonpharmacological recommendations in their treatment guidelines [6], due to the several limitation of the pharmacological treatments: possible side effects, poor adherence to therapy (half of the patients stop taking their medication at one year from starting) [7], or also the very slow actual development of new drugs for hypertension.

The use of nonpharmacological treatments, including, among others, the administration of nutraceutical supplements based on botanicals, has been growing in the recent years. Several plants, such as* Eucommia ulmoides* Oliv. [8],* Allium sativum* L. [9], and* Nigella sativa* L. [10], exert antihypertensive effects through different mechanisms and have been investigated for their clinical efficacy [11–13]. The main class of natural compounds responsible for the vascular effects is represented by polyphenols. Some grape juices and grape skin extracts rich in polyphenols exert endothelium-dependent relaxations in aortic rings [14]. Furthermore, polyphenol-rich sources, including extracts from red wines and green and black tea, determine endothelium-dependent relaxations in large arteries, arterioles, and veins that are prevented by competitive inhibitors of eNOS and guanylyl cyclase [14, 15]. In addition, red wine polyphenols have been shown to induce endothelium-dependent relaxation in porcine coronary artery rings [16]. Other studies demonstrated that polyphenol-rich red wine extracts suppress the angiotensin II-stimulated upregulation of several NADPH oxidase subunits including NOX1 and p22phox and the associated oxidative stress and hypertension [17]. Kane et al. [18] demonstrated that red wine polyphenols decrease angiotensin II-induced vascular expression of COX and the increased endothelium-derived contracting factors.

In addition, a chestnut wood extract, rich in ellagitannins, has been shown to exhibit an antioxidant activity and to produce a cardioprotective effect [19]. Polyphenols from terrestrial and marine plants exert several biological activities which result not only in a reduction of the blood pressure, but also in a protection from a wild variety of chronic diseases, including those affecting cardiovascular system [20, 21].

In this study we focused our attention on* Olea europaea* L. leaf extract (OEE) and on* Hibiscus sabdariffa* L. calyces extract (HSE). Phenolic compounds found in olive plant (*Olea europaea* L.), including hydroxytyrosol, oleuropein, flavonoids, chalcones, and tannins have been shown to exert many beneficial effects towards cardiovascular system [22]. Several studies have demonstrated that olive leaves exert antihypertensive, antiatherogenic, anti-inflammatory, hypoglycemic, and hypocholesterolemic effects [23]. In addition, olive leaf extract, or its main component oleuropein, shows protective effects in atherosclerosis [24], diabetes [25], hypertension [26], cardiotoxicity [27, 28], neurotoxicity [29], gastric lesions [30], and cancers [31].

Olive leaf extract exerts the hypotensive action through a direct activity on vascular smooth muscle where it determines a calcium antagonistic effect [32] and on endothelium [33]. A clinical trial confirmed the efficacy and safety of an olive leaf extract, named EFLA 943, in lowering systolic and diastolic blood pressures in subjects with stage-1 hypertension [34].

*Hibiscus sabdariffa* L. calices extracts have been reported to contain several polyphenols including flavonoids such as cyanidin 3-rutinoside, delphinidin 3-sambubioside, cyanidin 3-sambubioside, cyaniding-3-glucoside, delphinidin 3-glucoside, and hibiscus acid [35]. All these compounds of* Hibiscus sabdariffa* L. extract have been shown to attenuate atherosclerosis through several mechanisms such as the antioxidative activity [36], inhibition of LDL oxidation [37], and smooth muscle cell proliferation [38]. The hypotensive action of* Hibiscus sabdariffa* L. extract occurs, at least in part, through a diuretic activity, due to the modulation of the aldosterone action [39]. The latter action is mainly exerted by anthocyanins such as delphinidin-3-sambubioside and cyanidin-3-sambubioside, by phenylpropanoids such as chlorogenic acid, and, to a lesser extent, by flavonoids such as quercetin and rutin [39]. In addition, the anthocyanins delphinidin- and cyanidin-3-O-sambubiosides from* Hibiscus sabdariffa* L. are ACE competitive inhibitors [40]. HSE relaxes, in a concentration-dependent manner, aortic rings precontracted with KCl high concentration (80 mM) and phenylephrine (PE), thus it probably exerts a vasorelaxant activity through a mechanism involving voltage and receptor operated Ca^2+^ calcium channels (VOCC and ROCC, resp.) [41]. In addition, HSE exerts a direct activity towards vascular endothelium, producing a vasorelaxant effect via the activation of endothelium-derived nitric oxide/cGMP-relaxant pathway and the increased synthesis/release of endothelium-derived nitric oxide [41]. Several clinical trials confirmed hypotensive and antihypertensive effectiveness and safety of* Hibiscus sabdariffa* L. extract [42–44].

This study is aimed at investigating the cardiovascular effects of an* Olea europea* L. leaf extract (EFLA 943), of an* Hibiscus sabdariffa* L. calyces extract, and of a mixture of the two extracts, containing 86.67% of EFLA 943 and 13.33% of* Hibiscus sabdariffa* L. flower powder extract (13 : 2 w/w) in order to assess their cardiac and vascular synergistic or antagonistic activity. This EFLA 943/HSE ratio is commercially available as an adjuvant food supplement in the treatment of hypertension.

In this study, we have fully characterized both EFLA 943 and HSE extracts in their polyphenol composition, and we have evaluated the antioxidant and cytoprotective activity of both single extracts and their mixture in human umbilical vein endothelial cells (HUVECs). Moreover, the effect on cardiovascular system has been investigated by the evaluation of inotropic and chronotropic effects on guinea pig isolated left and right atria and of vasorelaxant effect in guinea pig isolated aortic strips. Comparison with ileum longitudinal smooth muscle was performed to discriminate the potential relaxant effect in vascular and nonvascular smooth muscle tissue.

## 2. Materials and Methods

### 2.1. Materials

*Olea europea* L. leaves extract (Benolea (EFLA 943)) (OEE) was supplied by Frutarom (Switzerland Ltd.). The extract, manufactured from the dried leaves of* Olea europaea* L., was obtained applying an ethanol (80% m/m) extraction procedure as previously described [45] and the product was purified by a patented procedure (US Patent 6024998) to remove undesired contaminants and residues. The solvent was subsequently removed resulting in a free flowing powder, containing 18−26% w/w oleuropein by HPLC analysis.

The* Hibiscus sabdariffa* L. flower powder extract (HSE) was supplied by Nutraceutica S.r.l. (via Idice 270/1 40050 Monterenzio, Bologna, Italy). Briefly dried calyces of* Hibiscus sabdariffa* L. were subjected to extraction with distilled water for 48 hrs. The extract was filtered and concentrated under reduced pressure and completely evaporated in a vacuum oven at a temperature not exceeding 40°C. The aqueous extract was dried using Freeze Dryer system. For deep information please visit Nutraceutica website (http://www.nutraceutica.it/).

The mixture of two extracts, containing 86.67% of EFLA 943 and 13.33% of* Hibiscus sabdariffa* L. flower powder extract, represents the active ingredient of a food supplement proposed as coadjuvant in the treatment of hypertension.

The extracts and the mixture were dissolved in water shortly before use.

3-(4,5-Dimethylthiazol-2-yl)-2,5-diphenyltetrazolium bromide (MTT), 2′,7′-dichlorodihydrofluorescein diacetate (DCFH-DA), and all other chemicals were purchased from Sigma-Aldrich (St. Louis, MO, USA). Acetonitrile (HPLC grade) was from VWR (Milano, Italy), and formic acid (98%–100%) was from Merck (Darmstadt, Germany). Deionized water was obtained from an Elix 10 water purification system from Millipore (Bedford, MA, USA).

### 2.2. Phytochemical Analysis

#### 2.2.1. Extraction of Phenolic Compounds

OEE and HSE (0.1 g) were subjected to extraction using 2 mL of a methanol/water solution (50 : 50, v/v) in a 15-mL centrifuge tube. The mixture was blended (Ultra-Turrax, IKA, Staufen, Germany) for 5 min and then centrifuged for 5 min at 2500 g. The hydroalcoholic extract was collected and the powders were reextracted with 2 mL of methanol/water solution. Finally, the hydroalcoholic fractions were combined, diluted up to 5 mL and filtered through 0.2 *μ*m regenerated cellulose filters (Schleicher & Schuell, Dassel, Germany).

#### 2.2.2. Liquid Chromatography Analysis of OEE and HSE

The analysis of OEE and HSE to detect phytochemicals was performed by a 1290 Infinity series liquid chromatography instrument (HPLC) equipped with a quaternary pump (Agilent Technologies, Waldbronn, Germany) coupled online with a UV-Vis detector. The separation of phenolic compounds was carried out on a reverse phase C18 100 Å Kinetex column (2.6 *μ*m, 100 × 3.00 mm I.D., Phenomenex, Torrance, CA, USA). Gradient elution was carried out with a solvent system of water/formic acid (100 : 0.5 v/v) as mobile phase A and acetonitrile as mobile phase B; the total run-time was 30 minutes and the gradient elution was as follows: from 0 to 5 min solvent B increased from 5% to 15%, at 10 min solvent B reached 25%, at 23 min solvent B reached 50%, and finally at 28 min solvent B was 100%; at 30 min 5% solvent B was restored. The column was thermostated at 30°C and equilibrated for 5 min prior to each analysis. An injection volume of 2.5 *μ*L and a flow rate of 0.7 mL min^−1^ were used. The chromatograms were monitored at three wavelengths (280, 320, and 345 nm) characteristic for a high number for aromatic compounds. Each wavelength was suitable for each group of compounds: 280 nm was used for secoiridoids, 320 nm for hydroxycinnamic acids, and 345 nm for flavones and flavonols.

For the structural elucidation and the detection of other compounds such as hibiscus acid, a typical compound present in HSE, the HPLC system was coupled online to a triple quadrupole mass spectrometer detector (QqQ, 6420 Triple Quad/LC MS, Agilent Technologies) equipped with a TurboIonSpray source operating in negative-ion mode. The declustering potential (DP), collision energy (CE), and focusing potential (FP) were optimized for oleuropein and vanillic acid. TurboIonSpray source of QqQ setting was as follows: capillary voltage −3500 V; nebulizer gas (N2) 50 (arbitrary units); curtain gas (N2) 12 (arbitrary units); collision gas (N2) 4 (arbitrary units); focusing potential −200 V; entrance potential 10 V; drying gas (N2) heated to 250°C and introduced to a flow rate of 12 mL min^−1^. Full-scan data acquisition was performed scanning from* m/z* 100 to 800 in profile mode and using a cycle time of 2 s with a step size of 0.1 *μ*m and a pause between each scan of 0.002 s; dwell time was set at 0.016 s.

In order to quantify through UV-Vis detector the amount of phenolic compounds in the hydroalcoholic OE extract, calibration curves were prepared with the available standards: oleuropein, verbascoside, quercetin-3-*O*-rhamnosyl-glucoside (rutin), and apigenin. The other compounds, for which no commercially standards were available, were tentatively quantified on basis of the other compounds bearing similar structures.

A calibration curve (*r* ^2^ > 0.99) of vanillic acid was also obtained to quantify hibiscus acid by ESI-QqQ-MS detector.

### 2.3. Animals

Guinea-pigs (males and females, 300–400 g) obtained from Charles River (Calco, Como, Italy) were housed in a controlled environment with a 12:12-h light-dark cycle at 22°C and provided with chow diet and water* ad libitum*. All animals used in this study were housed and treated according to the directives on the protection of animals used for scientific purposes (Directive 2010/63/EU of the European Parliament and of the Council) and the WMA Statement on Animal Use in Biomedical Research. All procedures followed the guidelines of animal care and were approved by the Ethics Committee of the University of Bologna (Bologna, Italy). The animals were sacrificed by cervical dislocation; the organs were immediately removed and used as below described.

#### 2.3.1. Atrial Preparations

The removed heart was washed by perfusion through the aorta with oxygenated Tyrode solution containing (mM): NaCl 136.9; KCl 5.4; CaCl_2_ 2.5; MgCl_2_ 1.0; NaH_2_PO_4_ *x*H_2_O 0.4; NaHCO_3_ 11.9; and glucose 5.5. The physiological salt solution (PSS) was buffered at pH 7.4 by saturation with 95% O_2_–5% CO_2_ gas, and the temperature was maintained at 35°C. The following isolated guinea-pig heart preparations were used: spontaneously beating right atria and left atria driven at 1 Hz. For each preparation, the entire left and right atria were dissected from the ventricles, cleaned of excess tissue, and hung vertically in a 15 mL organ bath containing PSS continuously bubbled with 95% O_2_–5% CO_2_ at 35°C, pH 7.4. The contractile activity was recorded isometrically by means of force transducer (FT 0.3, Grass Instruments Corporation, Quincy, MA, USA) using Power Lab software (ADInstruments Pty Ltd., Castle Hill, Australia). The left atria were stimulated by rectangular pulses of 0.6–0.8 ms duration and about 50% threshold voltage through two platinum contact electrodes in the lower holding clamp (Grass S88 Stimulator). The right atria were in spontaneous activity. After the tissues were beating for several min, a length-tension curve was determined, and the muscle length was maintained at the value eliciting 90% of maximum contractile force observed at the optimal length. A stabilization period of 45–60 min was allowed before the atria were challenged by various agents. During the equilibration period, the bathing solution was changed every 15 min and the threshold voltage was ascertained for the left atria. Atrial muscle preparations were used to examine the inotropic and chronotropic activity of the extracts and mixture (0.01–10 mg/mL), dissolved in PSS. During the generation of cumulative concentration-response curves, the next higher concentration of different extracts or mixture was added only after the preparation reached a steady state. All data are reported as means ± SEM. The EC_50_ values were calculated from concentration-response curves [46].

#### 2.3.2. Aortic Strips and Ileum Longitudinal Smooth Muscle (GPLSM) Preparations

The thoracic aorta and ileum were placed in Tyrode solution containing (mM): NaCl, 118; KCl 4.75; CaCl_2_ 2.54; MgSO_4_ 1.20; KH_2_PO_4_ 1.19; NaHCO_3_ 25; and glucose 11, equilibrated with 95% O_2_–5% CO_2_ at pH 7.4. The vessel was cleaned of extraneous connective tissue. Two helicoidal strips (10 mm × 1 mm) were cut from each aorta beginning from the end most proximal to the heart. Vascular strips were then tied with surgical thread (6–0) and suspended in a jacketed tissue bath (15 mL) containing aerated PSS at 35°C. Aortic strips were secured at one end to plexiglass hooks and connected via the surgical thread to a force displacement transducer (FT 0.3, Grass Instruments Corporation) for monitoring changes in isometric contraction. Aortic strips were subjected to a resting force of 1 g. The intestine was removed above the ileocaecal junction. GPILSM segments of 2 cm length were mounted under a resting tension of 300–400 mg. Strips were secured at one end to a force displacement transducer (FT 0.3, Grass Instruments Corporation) for monitoring changes in isometric contraction and washed every 20 min with fresh PSS for 1 h. After the equilibration period, guinea-pig aortic and GPLSM strips were contracted by washing in PSS containing 80 mM KCl (equimolar substitution of K^+^ for Na^+^). When the contraction reached a plateau (about 45 min) different concentrations of the extracts and mixture (0.01–10 mg/mL) were added cumulatively to the bath allowing for any relaxation to obtain an equilibrated level of force. All data are reported as means ± S.E.M. The IC_50_ were calculated from concentration-response curves [46].

### 2.4. Antioxidant and Cytoprotective Activities

#### 2.4.1. Cell Culture and Treatments

HUVECs were cultured as previously reported [47]. Briefly, cells were plated on gelatin-coated multiwell plates and maintained in complete medium M200 containing 10% FBS and growth factors at 37°C with 5% CO_2_. Cells from passages 3 to 6 were used. Cells at 80% confluence were treated for 24 h with different concentrations (0.05–100 *μ*g/mL) of OEE and HSE or a 13 : 2 w/w mix of the two and used for further analysis.

#### 2.4.2. Determination of Cell Viability

Viability of control and treated cells was measured using the MTT assay as previously reported [48]. For the flow cytometry analysis the cells were double labelled with Annexin V conjugated to Phycoerythrin (Annexin V-PE) and 7-amino-actinomycin D (7 AAD) and immediately analysed on a GuavaEasyCyte flow cytometer (Guava Technologies, Hayward, CA) in accordance with the manufacturer's instructions as reported in [49]. The percentage of viable cells was reported with respect to the total number of cells.

#### 2.4.3. Detection of Intracellular Reactive Oxygen Species

The formation of reactive oxygen species (ROS) was evaluated using a fluorescent probe, DCFH-DA, as previously reported [50]. Briefly, controls and treated cells were washed with PBS and then incubated with 5 *μ*M DCFH-DA in PBS for 30 min. After DCFH-DA removal, the cells were incubated with 100 *μ*M H_2_O_2_ for 30 min. Cell fluorescence from each well was measured using a microplate spectrofluorometer (*λ*_ex_ = 485 nm and *λ*_em_ = 535 nm). Intracellular antioxidant activity was expressed as the percentage of inhibition of intracellular ROS produced by H_2_O_2_ exposure.

#### 2.4.4. Determination of Cytoprotective Effect

Cytoprotection against H_2_O_2_ induced cell damage was assessed using the MTT assay as previously reported [19]. Control and treated cells were exposed to 150 *μ*M H_2_O_2_ in PBS for 3 h after which cells were changed to a fresh culture medium. MTT was added to the medium at the final concentration of 0.5 mg/mL and incubated for 4 h at 37°C. DMSO was added to dissolve the formazan crystals and the absorbance was measured at 595 nm using a microplate reader VICTOR3 V Multilabel Counter. Data were expressed as percentage of viable cells with respect to controls times. For the flow cytometry analysis the cells were double labelled with Annexin V conjugated to Annexin V-PE and 7 AAD and analysed on a GuavaEasyCyte flow cytometer in accordance with the manufacturer's instruction.

### 2.5. Statistical Analysis

Data on atria and on vascular and nonvascular smooth muscle were analyzed by the Student's *t*-test and presented as means ± S.E.M. [46]. *P* value less than 0.05 has been considered significant. The potency of different extract and its mixture, defined as EC_50_, EC_30_, and IC_50_ were calculated from concentration-response curves (Probit analysis using Litchfield and Wilcoxon [46] or GraphPad Prism [51, 52] software).

Data from HUVEC cultures are means ± S.D. and were analyzed by one-way analysis of variance

(ANOVA) followed by Dunnett's test, and *P* < 0.05 has been considered significant.

## 3. Results

### 3.1. Characterization of* Olea europea* L. Leaf and* Hibiscus sabdariffa* L. Flower Extracts

A complete characterization of OEE and HSE was realized by HPLC coupled to a UV-Vis and QqQ-Ms detector. When reference standard was not available, a tentative identification of compounds was made on the basis of spectroscopic properties, molecular weight, and the search of the main [M-H]-ion together with the interpretation of its fragments (Table 1).

**Figure 1:**
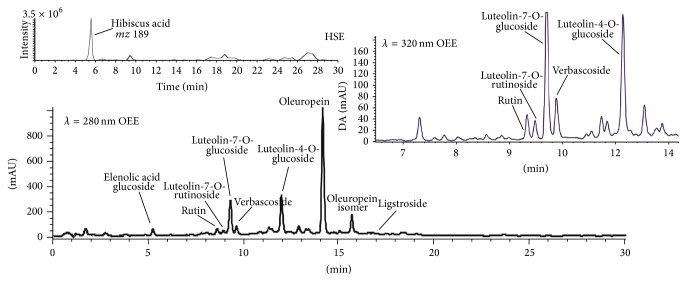
Phenolic profile of OEE at *λ* = 280 and 320 nm and extracted ion chromatography of hibiscus acid. The analysis of phenolic compounds was performed by liquid chromatography coupled online with a UV-Vis detection and a triple quadrupole mass spectrometer as reported in Methods section.

Hibiscus acid was detected using mass spectrometry by matching the information of molecular ion at* m/z* 189 in negative mode (Figure 1). Elenolic acid glucoside was tentatively identified by UV spectra at 240 nm but not corroborated by mass spectra. The identification of hydroxy oleuropein and oleuropein isomer was corroborated by detection of the molecular ion (at* m/z* 555 and 539, resp.) and their aglycone fragment at* m/z* 377. Verbascoside was identified by molecular ion at* m/z* 623 and various fragments (*m/z* 461, 315) that are in accordance with the fragmentation pathway. Luteolin-4-O-rutinoside, luteolin-7-*O*-glucoside, and luteolin-4-*O*-glucoside were detected by an intense molecular ion at* m/z* 447 and the diagnostic fragment at* m/z* 285 of luteolin derivatives.

To quantify phenolic compounds, calibration curves were obtained for each standard with high linearity (*r* ^2^ = 0.99) by plotting the standard concentration as a function of the peak area obtained from HPLC-UV. Hibiscus acid was expressed as equivalents of vanillic acid, not present in the extract, but used as standard for the quantification of this compound. Only hibiscus acid was identified and quantified in HSE, probably due to the extraction process used by the producer that washed or degraded the phenolic acids and flavonoids commonly reported in other hibiscus extracts. Phenolic compounds characterized in OEE include oleuropein and its isomers, luteolin-4-O- and luteolin-7-O-glucoside, luteolin-4-O-rutinoside, ligstroside, elenolic acid glucoside, verbascoside, and rutin (Figure 1).

The four major compounds found in olive leaf powder extract were oleuropein and oleuropein isomer and two glucoside isomers of luteolin (215, 51, and 5.8 and 4.2 mg g^−1^, resp.). Other minor compounds, detected in less quantity were hydroxy oleuropein, ligstroside, verbascoside, and rutin as reported in Table 1. All the compounds reported are typically present in olive leaf as previously reported [53, 54].

### 3.2. Effects of OEE, HSE, and Their Mixture on Guinea-Pig Isolated Cardiac and Smooth Muscle Tissues

The effects of single extracts and mixture were derived on guinea-pig isolated left and right atria to evaluate their inotropic and chronotropic effects, respectively, and on K^+^-depolarized (80 mM) guinea-pig vascular (aorta) and nonvascular ileum longitudinal smooth muscle (GPILSM) strips to assess calcium antagonist activity. Tested samples were checked at increasing doses to evaluate the percent decrease of developed tension on isolated left atrium driven at 1 Hz (negative inotropic activity), the percent decrease in atrial rate on spontaneously beating right atrium (negative chronotropic activity), and the percent inhibition of calcium-induced contraction on K^+^-depolarized aortic strips and GPILSM (vascular and nonvascular relaxant activity, resp.).

#### 3.2.1. Guinea-Pig Left and Right Atria

Data relative to inotropic and chronotropic activities of OEE, HSE, and their mixture were reported in Table 2. Both extracts (1 mg/mL) produced negative inotropic effect in left atria driven at 1 Hz (68 ± 2.4% and 76 ± 0.9%, resp.). On the contrary, OEE potency was 1.9 times higher than that of HSE extract (EC_50_ = 0.14 mg/mL (c.l. 0.10–0.18) and EC_50_ = 0.27 mg/mL (c.l. 0.21–0.35), resp.). The mixture exerts an intrinsic activity, slightly lower than that exerted by the single extracts; however its potency was not significantly different from the potency of OEE (EC_50_ = 0.16 mg/mL (c.l. 0.12–0.020) and EC_50_ = 0.14 mg/mL (c.l. 0.10–0.18), resp.) but it was 1.7 times higher than that of HSE (EC_50_ = 0.16 mg/mL (c.l. 0.12–0.20) and EC_50_ = 0.27 mg/mL (c.l. 0.21–0.35) resp.). Both extracts did not show negative chronotropic activity. As shown in Table 2 intrinsic activity was less than 50% (37 ± 2.4% and 46 ± 0.7%, resp.) at the maximal dose tested (10 mg/mL for OEE and 1 mg/mL for HSE, resp.). On the contrary, the mixture (1 mg/mL) revealed a negative chronotropic intrinsic activity (84 ± 2.0% at 10 mg/mL) with a potency 7.6 time higher than the inotropic negative potency (EC_30_ = 1.21 mg/mL (c.l. 1.10–1.33) and EC_50_ = 0.16 mg/mL (c.l. 0.12–0.20), resp.).

#### 3.2.2. Guinea Pig Smooth Muscle

Both extracts reduced in a dose-dependent manner the contraction induced by 80 mM K^+^ on the vascular smooth muscle (Table 3). Both OEE and HSE had similar intrinsic activity at the same maximal concentration (10 mg/mL) analyzed. On the contrary, the vasorelaxant potency of OEE was 6.7 times higher than that of HSE (IC_50_ = 5.15 mg/mL (c.l. 4.68–5.59) and IC_50_ = 6.63 mg/mL (c.l. 6.34–6.92), resp.). The mixture revealed an intrinsic activity lower than that of the single extracts at the same maximal concentration tested. In agreement with data on negative inotropic effect, the potency of the mixture was not significantly different from those of OEE (IC_50_ = 5.89 mg/mL (c.l. 5.56–6.25) and IC_50_ = 5.15 mg/mL (c.l. 4.68–5.59), resp.). Moreover, the vasorelaxant effect of mixture is about 1.1 times higher than that of the HSE (IC_50_ = 5.89 mg/mL (c.l. 5.56–6.25) and IC_50_ = 6.63 mg/mL (c.l. 6.34–6.92), resp.). On the contrary, only OEE demonstrated intrinsic activity in nonvascular smooth muscle (Table 3). The OEE was 6.7 times more potent in the nonvascular (GPLSM) than in the vascular smooth muscle (aorta) (IC_50_ = 0.77 mg/mL (c.l. 0.34–1.01) and IC_50_ = 5.15 mg/mL (c.l. 4.68–5.59), resp.) evidencing its selectively. In GPILSM, the mixture revealed an intrinsic activity not significantly different from these of OEE (Table 2) with a potency 1.8 times lower than that of OEE alone (IC_50_ = 1.39 mg/mL (c.l. 1.13–1.54) and IC_50_ = 0.77 mg/mL (c.l. 0.34–1.01), resp.) suggesting that the addition of a little amount of HSE to OEE is able to reduce the selectivity toward the nonvascular tissue.

### 3.3. Antioxidant and Cytoprotective Effects in Cultured HUVECs

Since olive leaves and hibiscus flower extracts are rich in phenolic compounds, we have investigated the ability of the extracts to protect cultured HUVECs from oxidative stress.

**Figure 2:**
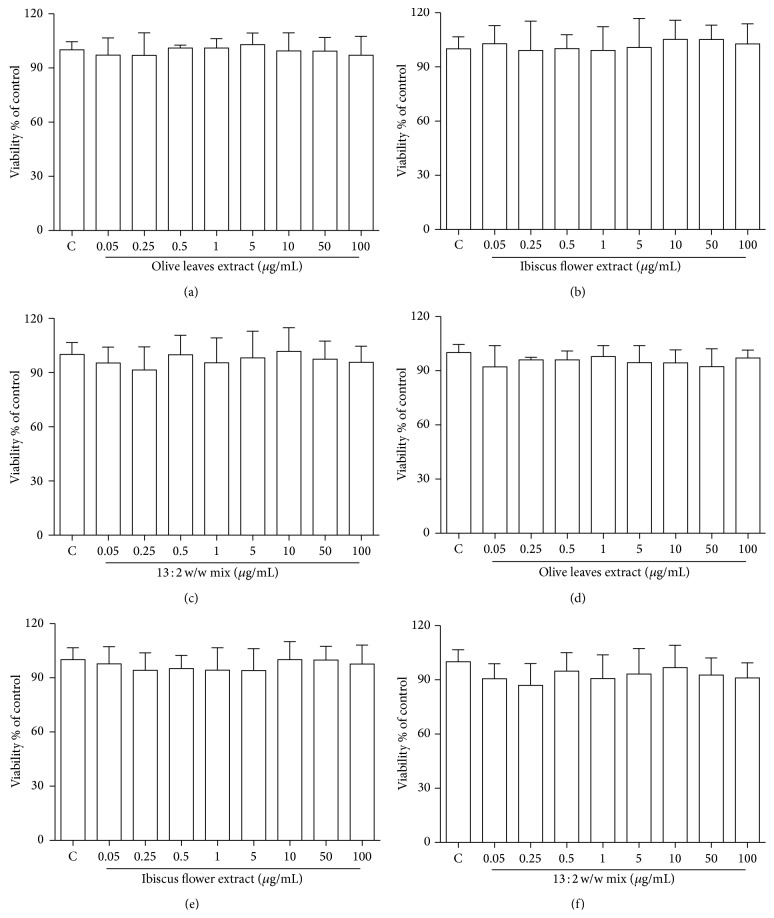
Cell viability of HUVECs treated with OEE, HSE, and their mixture. HUVECs were treated as described in Methods section. Cell viability was analysed by the MTT test as reported in the Methods section ((a), (b), and (c)). Cell viability was analysed by flow cytometry. Cells were double-labelled with Annexin V-PE 7 AAD and analyzed by a Guava EasyCyte flow cytometer ((d), (e), and (f)). Data are reported as means ± S.D. of four independent experiments.

Figure 2 shows, by both MTT (Figures 2(a), 2(b), and 2(c)) and flow cytometry analysis (Figures 2(d), 2(e), and 2(f)), that OEE, HSE, and their mixture did not exert any toxic effect on cultured HUVECs on a wide range of concentrations (0.05–100 *μ*g/mL).

**Figure 3:**
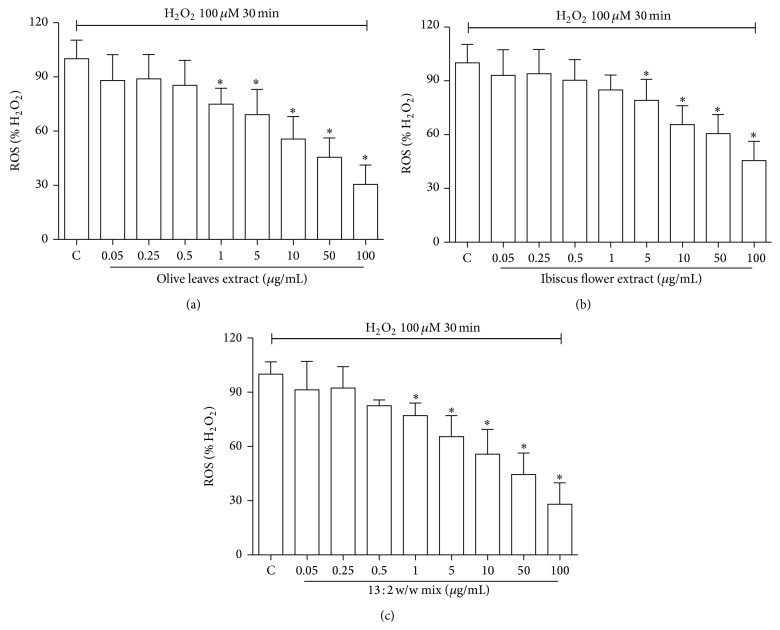
Effect of OEE, HSE, and their mixture on intracellular ROS production. HUVECs were treated with OEE (a), HSE (b), and their mixture (c) (0.05–100 *μ*g/mL) for 24 h; oxidative damage was then induced exposing the cells to 100 *μ*M H_2_O_2_ for 30 min and intracellular ROS were determined using the peroxide-sensitive fluorescent probe DCFH-DA as described in Methods section. Data are expressed as percent of control cells treated with H_2_O_2_. Values represent means ± S.D. of four independent experiments. ^*^ *P* < 0.05 with respect to H_2_O_2_-treated cells.

ROS level measurement revealed that ROS production was significantly reduced in extract treated cells after 24 h in a dose-dependent manner.  Figure 3(a) shows significant decrease in ROS production, as detected by DCFH-DA assay that was observed in both OEE and HSE treated HUVECs following exposure to H_2_O_2_ (Figures 3(a) and 3(b)), with OEE exhibiting the highest effect, with a significant decrease in ROS production at 1 *μ*g/mL, while HSE evidenced a significant effect only at 5 *μ*g/mL.

The mix revealed a significant antioxidant activity at 1 *μ*g/mL (Figure 3(c)). The calculated IC_50_ values were 17.34 *μ*g/mL and 25.04 *μ*g/mL for OEE and HSE, respectively. Interestingly, the IC_50_ value of the mixture (17.00 *μ*g/mL) was lower, although not significantly, than the OEE one.

**Figure 4:**
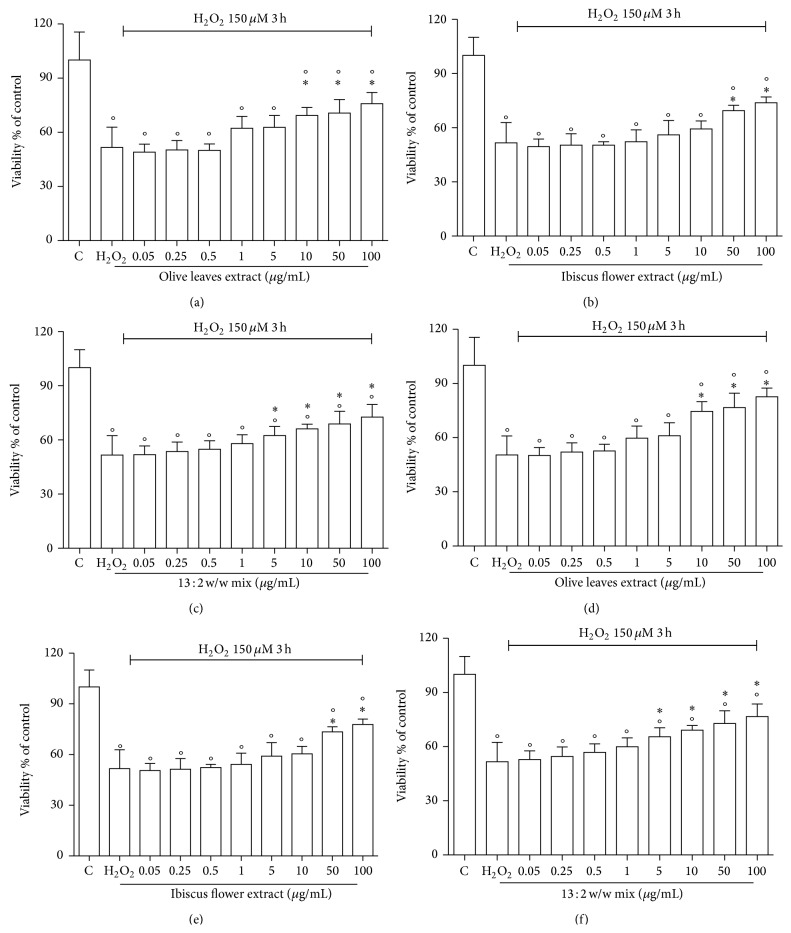
Effect of OEE, HSE, and their mixture on cell viability in HUVECs exposed to H_2_O_2_. HUVECs were treated with OEE, HSE, and their mixture (0.05–100 *μ*g/mL) for 24 h; oxidative damage was then induced exposing the cells to 150 *μ*M H_2_O_2_ for 3 h and cellular damage was assessed by both MTT assay ((a), (b), and (c)) and flow-cytometry analysis ((d), (e), and (f)). Data are reported as percent cell viability in comparison to control cells. Each bar represents the mean ± S.D. of four independent experiments. ^*^ *P* < 0.05 with respect to H_2_O_2_-treated cells, °*P* < 0.05 with respect to control cells.

Incubation of HUVECs with 150 *μ*M H_2_O_2_ for 3 hours caused a significant decrease in cell viability (Figure 4), as detected by MTT reduction assay. OEE revealed a significant cytoprotective effect at 10 *μ*g/mL (Figure 4(a)) while HSE at 50 *μ*g/mL (Figure 4(b)). The mixture was able to significantly protect cells against H_2_O_2_ induced damage at 5 *μ*g/mL (Figure 4(c)), suggesting a synergistic effect in cytoprotection. Flow cytometry analysis confirmed MTT data (Figures 4(d), 4(e), and 4(f)).

## 4. Discussion

OEE and HSE are rich in phytochemicals, the effects of which have been widely investigated. They have been used in the human diet as an extract, a herbal tea, and a powder. Their bioactive compounds have been demonstrated to have antioxidant, antihypertensive, antiatherogenic, anti-inflammatory, hypoglycemic, and hypocholesterolemic properties [23, 55].

In the present paper, we have focused the attention on the potential application of OEE and HSE in the prevention/counteraction of hypertension, a pathological condition affecting a large number of populations. Hypertension management is a primary factor in the prevention of pathological events as heart attacks, cerebrovascular diseases, and renal failure.

Conventional approach relies on drugs with different biological targets. Oral antihypertensive drugs associated to exercise, lifestyle, and dietary modification are essential approaches for hypertension therapy [1]. However, hypertension control is not always satisfactory [56], and nowadays some patients have turned to natural substances [57], which, as complementary therapy, appear to be promising in reducing blood pressure and relieving signs and symptoms in hypertensive patients [58].

*Olea europea* L. and* Hibiscus sabdariffa* L. extracts are known to exert antihypertensive effects [23, 55]. In this paper using both cultured HUVECs and guinea-pig left and right atria and vascular smooth muscle we have demonstrated their antioxidant and cytoprotective effects and their ability to modulate cardiac inotropy and chronotropy together with their relaxant activity on vascular smooth muscle.

*Olea europea* L. is an evergreen tree typical of the Mediterranean region. Not only olive oil, but also olive leaves revealed antihypertensive properties mainly attributed to the presence of polyphenols (as oleuropein and analogues), due to their L-type calcium channels blocking ability [26, 32]. Oleuropein is therefore a functional analogue of well-known L-Type calcium channel entry blockers such as nifedipine.

Interesting studies on oleuropein and olive polyphenols metabolism and bioavailability have been recently conducted. Suárez et al. identified the presence of oleuropein metabolites in a simulated in vitro model of gastrointestinal digestion [59]. Data on oleuropein bioavailability have been reported by García-Villalba et al., who demonstrated that many phenolic metabolites are detectable in urine sample of women supplemented with 250 mg of an oleuropein-rich olive leaves extract. Moreover the authors showed that some metabolites such as hydroxytyrosol glucuronide are present in urine at micromolar concentration [60]. Absorption of biologically active compounds has been demonstrated after chronic administration of olive leaves extract to rats and mice [61]. de Bock et al. published data showing that oleuropein is bioavailable in the parental form after oral ingestion of an olive leaves extract with a peak plasma concentration in the order of ng/mL [62]. Possible mechanisms have been proposed for oleuropein and oleuropein metabolites absorption; Manna et al. proposed that oleuropein-glycoside may diffuse through the lipid bilayer of the epithelial cell membrane and be absorbed via a glucose transporter. Moreover additional mechanisms for oleuropein-glycoside absorption are potential via the paracellular or transcellular passive diffusion [63].

OEE concentrations used in “in vitro” assays on HUVEC cells are representative of oleuropein and oleuropein metabolite concentrations found in plasma after ingestion of olive leaf extracts.

Data obtained on cardiac guinea pig isolated tissues show that OEE exerted negative inotropic effect on left atrium driven at 1 Hz, without negative chronotropic effect on spontaneous beating right atrium in contrast with nifedipine, which present both negative inotropic and chronotropic effects [64]. Differently from nifedipine, OEE is presumably selective for the Cav1.2 subunit, known as cardiac isoform, widely expressed in the cardiovascular system where it regulates the vascular tone and cardiac inotropy, without effect on Cav1.3 subunit predominantly expressed in neurons and in cardiac pacemaker cells responsible for the chronotropic effect [65]. Similarly to nifedipine, OEE reduced the potassium (80 mM) induced contraction on guinea-pig aorta strips. As far as the vascular and not vascular smooth muscle relaxant activity, OEE is similar to nifedipine. Nifedipine selectivity for vascular smooth muscle over the cardiac parameters has so far allowed its use and that of subsequent 1,4-DHP's generations in the treatment of hypertension [66, 67]. Differently from nifedipine, in OEE, the inotropic effect prevails over the effects on smooth muscle.

Olive (*Olea europaea*) leaf extract, at the dosage regimen of 500 mg twice daily, was similarly effective in lowering systolic and diastolic blood pressures in subjects with stage-1 hypertension as Captopril, given at its effective dose of 12.5–25 mg twice daily [34].

The OEE concentrations used in “ex vivo” assays on isolated tissue are in line with doses reported in human intervention studies with oleuropein enriched nutritional supplements [34, 68]

*Hibiscus sabdariffa* L. is a native shrub of tropical Africa. Dried flowers are used to prepare an infusion with soothing and refreshing properties [69]. The hypotensive activity is partly due to its vascular smooth muscle vasorelaxant properties, as shown in Table 3. HSE share common inotropic and chronotropic effects with OEE, but with less potency. HSE, similarly to OEE, shows relaxant effects on the vascular but not on the nonvascular smooth muscle. It has been recently reported that diuresis and inhibition of the angiotensin I-converting enzyme are less important mechanisms to explain the beneficial actions than those related to the antioxidant, anti-inflammatory, and endothelium-dependent effects [70].

These effects could be ascribed to hibiscus acid, the main phytochemical present in hibiscus calyces [71]; moreover hibiscus flowers are rich in anthocyanosides [72], known to increase microvessels resistance and reduce the permeability and damage [73, 74].

Recently Fernández-Arroyo et al. published data regarding bioavailability and metabolism of* Hibiscus sabdariffa* L. organic acids and polyphenols after oral ingestion in rats. Through HPLC-ESI-TOF-MS analysis, the authors demonstrated that phenolic acids were detectable in plasma without any structural modification; most flavonols were found as quercetin or kaempferol glucuronide conjugates. After oral administration, hibiscus acid, hibiscus acid hydroxyethyl ester, and the metabolite hydroxycitric acid reached high concentrations in plasma, contributing to micromole amounts of organic acids in plasma [75].

To demonstrate that a multifaceted and likely synergistic mechanism accounts for the hypotensive action of OEE and HSE mixture, in vitro study on HUVECs has been performed to evaluate the antioxidant and cytoprotective effects of the two extracts and mixture towards endothelial cells which exert a central role in the regulation of blood pressure.

OEE and HSE revealed cytoprotective and antioxidant properties and OEE extract showed the highest effect. Several authors demonstrated the synergistic effect of many plant extracts administered in polyherbal formulations [75–78]. In this study we have demonstrated that the mixture of the two extracts exerts higher cytoprotective and antioxidant activities than each single extract. Both of these functions are critically involved in cardiovascular protection and hypertension control as clearly reported in the literature. Oxidative stress is also markedly increased in hypertensive patients. If oxidative stress is indeed a cause of hypertension, then antioxidants should have beneficial effects on hypertension control and reduction of oxidative damage should result in a reduction in blood pressure [79].

Another key factor in evaluating the effect of OEE and HSE on the endothelium is their capacity to react to different injuries [80]. Tissue damage at the vascular wall and inflammation leads to stress in the endothelium, so studies on the cytoprotective effect at the endothelium level are of great importance. Some factors have been identified which impair vascular endothelium function, both by their direct effects on the vascular vasomotor capacity, or by influencing cellular regulators, such as inflammatory mediators (ICAM, VCAM). What we currently know about the etiopathogeny of atherosclerosis is that it is a chronic oxidative stress-related inflammatory disease. This study performed on the antioxidant and cytoprotective effects of OEE and HSE throws light on this important point, delineating the cellular mechanism in vascular health protection.

The mixture showed an intrinsic inotropic activity (left atrium) lower than each single extracts, while it exerted negative chronotropic effect; globally the inotropic effect is lower than chronotropic effect, in contrast to nifedipine, characterized by a higher chronotropic than inotropic effect [64]. Despite the low percentage in the mixture, HSE was able to strengthen the effect of OEE, revealing a synergistic cardiac and vascular protective effect. However, even if the amounts of the other phytochemicals are very low, the percentage of HSE is sufficient to reduce the effects of OEE on nonvascular tissue, directing the mixture selectivity towards the vascular tissue.

On the whole, these findings show that the combined treatment of cultured cells, atria, and vascular tissue with the mixture revealed high cardiac and vascular protective activities that, in our specific experimental conditions, were higher than that observed for the most powerful component (OEE).

In conclusion OEE and HSE mixture has a huge potential for pharmaceutical and nutraceutical application, thanks to the synergistic effects of the single phytochemicals.

## Figures and Tables

**Table 1:** UV absorption bands, mass spectrometry, and quantitative analysis of hibiscus acid and phenolic compounds in HSE and OEE.

Compound	*λ*_max⁡_ (nm)	Mass spectrometry data			Quantitative data (mg g^−1^)	
		MW	[M-H]^−^	Main fragments	OEE	HSE
Hibiscus acid^1^	—	190	189		—	139.2 ± 0.47
Secoiridoids						
Hydroxyoleuropein^2^	280	556	555	539, 377, 197	8.96 ± 0.42	—
Elenolic acid glucoside^2^	240	404	—	—	23.65 ± 0.79	—
Oleuropein^2^	280	540	539	377, 197	215.1 ± 1.64	—
Oleuropein isomer^2^	280	540	523	377, 197	51.09 ± 0.35	—
Ligstroside^2^	280	524	523	361, 191	11.03 ± 0.04	—
Hydroxycinnamic acids						
Verbascoside^3^	320	624	623	461, 315, 135	4.67 ± 0.44	—
Flavonols						
Rutin^4^	345	610	609	301, 179	0.69 ± 0.06	—
Flavones						
Luteolin-7-*O*-rutinoside^5^	345	594	593	447, 285	0.74 ± 0.04	—
Luteolin-7-*O*-glucoside^5^	345	448	447	285	5.83 ± 0.24	—
Luteolin-4-*O*-glucoside^5^	345	448	447	285	4.20 ± 0.12	—

Hibiscus acid and phenolic compounds quantified as: ^1^vanillic acid mg/g; ^2^oleuropein mg/g; ^3^verbascoside mg/g; ^4^rutin mg/g; ^5^apigenin mg/g. Values are means ± S.D. (*n* = 3).

**Table 2:** Inotropic and chronotropic effects of OEE, HSE, and their mixture.

Extract	Left atrium			Right atrium		
	Negative inotropy			Negative chronotropy		
	Activity^a^ (M ± SEM)	EC_50_^b^ (mg/mL)	95% conf. lim.	Activity^c^ (M ± SEM)	EC_30_^b^ (mg/mL)	95% conf. lim.
OEE	68 ± 2.4	0.14	0.10–0.18	37 ± 2.4		
HSE	76 ± 0.9	0.27	0.21–0.35	46 ± 0.7^d^		
Mixture	60 ± 1.4	0.16	0.12–0.20	84 ± 2.0	1.21	1.10–1.33

^a^Decrease in developed tension on isolated guinea-pig left atrium at 1 mg/mL concentration, expressed as percent changes from the control (*n* = 5-6). The left atria were driven at 1 Hz. The 1 mg/mL concentration gave the maximum effect for most compounds. ^b^Calculated from concentration-response curves (Probit analysis by Litchfield and Wilcoxon [46] with *n* = 6-7). When the maximum effect was <50%, the EC_50_ ino., EC_30_ chrono., values were not calculated. ^c^Decrease in atrial rate on guinea-pig spontaneously beating isolated right atrium 10 mg/mL concentration, expressed as percent changes from the control (*n* = 7-8). The 10 mg/mL concentration gave the maximum effect for most compounds. Pretreatment heart rate ranged from 165 to 190 beats/min. ^d^At 1 mg/mL concentration.

**Table 3:** Relaxant activity of OEE, HSE, and their mixture on K^+^-depolarized guinea pig smooth muscle.

Extract	Aorta			GPILSM		
	Activity^a^ (M ± SEM)	IC_50_^b^ (mg/mL)	95% conf. lim.	Activity^a^ (M ± SEM)	IC_50_^b^ (mg/mL)	95% conf. lim.
OEE	90 ± 1.1	5.15	4.68–5.59	90 ± 1.7	0.77	0.34–1.01
HSE	93 ± 1.4	6.63	6.34–6.92	21 ± 0.7^c^		
Mixture	70 ± 3.6	5.89	5.56–6.25	90 ± 1.2	1.39	1.13–1.54

^a^Percent inhibition of calcium-induced contraction on K^+^-depolarized (80 mM) guinea-pig aortic strips at 10 mg/mL concentration. The 10 mg/mL concentration gave the maximum effect for most compounds, respectively. ^b^Calculated from concentration-response curves (Probit analysis by Litchfield and Wilcoxon [46] with *n* = 6-7). When the maximum effect was <50%, the IC_50_ values were not calculated. ^c^At 5 mg/mL concentration.
